# Uric Acid and the Prediction Models of Tumor Lysis Syndrome in AML

**DOI:** 10.1371/journal.pone.0119497

**Published:** 2015-03-16

**Authors:** A. Ahsan Ejaz, Negiin Pourafshar, Rajesh Mohandas, Bryan A. Smallwood, Richard J. Johnson, Jack W. Hsu

**Affiliations:** 1 Division of Nephrology, Hypertension and Transplantation, North Florida/South Georgia Veterans Health System, Gainesville, Florida, United States of America; 2 Nephrology & Hypertension Section, Department of Veterans Affairs Medical Center, North Florida/South Georgia Veterans Health System, Gainesville, Florida, United States of America; 3 Department of Epidemiology, College of Public Health and Health Professions and the College of Medicine, University of Florida, Gainesville, Florida, United States of America; 4 Division of Renal Diseases and Hypertension, University of Colorado Health Sciences Center, Gainesville, Florida, United States of America; 5 Division of Hematology and Oncology, University of Florida, Gainesville, Florida, United States of America; IPK, GERMANY

## Abstract

We investigated the ability of serum uric acid (SUA) to predict laboratory tumor lysis syndrome (LTLS) and compared it to common laboratory variables, cytogenetic profiles, tumor markers and prediction models in acute myeloid leukemia patients. In this retrospective study patients were risk-stratified for LTLS based on SUA cut-off values and the discrimination ability was compared to current prediction models. The incidences of LTLS were 17.8%, 21% and 62.5% in the low, intermediate and high-risk groups, respectively. SUA was an independent predictor of LTLS (adjusted OR 1.12, CI95% 1.0–1.3, p = 0.048). The discriminatory ability of SUA, per ROC curves, to predict LTLS was superior to LDH, cytogenetic profile, tumor markers and the combined model but not to WBC (AUCWBC 0.679). However, in comparisons between high-risk SUA and high-risk WBC, SUA had superior discriminatory capability than WBC (AUCSUA 0.664 vs. AUCWBC 0.520; p <0.001). SUA also demonstrated better performance than the prediction models (high-risk SUAAUC 0.695, p<0.001). In direct comparison of high-risk groups, SUA again demonstrated superior performance than the prediction models (high-risk SUAAUC 0.668, p = 0.001) in predicting LTLS, approaching that of the combined model (AUC 0.685, p<0.001). In conclusion, SUA alone is comparable and highly predictive for LTLS than other prediction models.

## Introduction

Tumor lysis syndrome (TLS) is a medical emergency, a consequence of cell lysis and the rapid release of intracellular contents into the blood stream and the potential for subsequent multiple organ damage leading to renal failure, cardiac arrhythmia, pulmonary edema and death. TLS, once reserved for high-bulk chemosensitive tumors, is increasingly being diagnosed in nontraditional settings due to the introduction of omnipotent drugs in clinical practice. Therefore, prediction of TLS and institution of prophylactic and therapeutic options are paramount to the favorable clinical outcomes for patients undergoing cancer treatment. The current prediction models of laboratory TLS (LTLS) in acute myeloid leukemia (AML) are based on white blood cell count (WBC), with or without lactate dehydrogenase (LDH), and specific cytogenetic abnormalities and karyotype complexity. None of the prediction models include serum uric acid (SUA). Recent experimental and clinical studies have demonstrated that SUA has prooxidative and pro-inflammatory properties via crystal dependent and independent mechanisms [1–2]. We have also demonstrated that SUA is an independent predictor of acute kidney injury (AKI) [3]. Given our findings, we wanted to investigate the discrimination ability of baseline SUA to predict TLS and also to compare it to the conventional prediction models.

## Materials and Methods

In this retrospective analysis, 183 consecutive patients with diagnosis of AML were identified from an oncology database at the University of Florida between 2000 and 2012. Data on patient demographics, comorbidities, tumor characteristics, cytogenetic abnormalities, gene mutations, tumor markers, laboratory parameters, prophylaxis and treatment regimen, length of hospital stay (LOS), and incidences of AKI and LTLS were extracted from electronic medical records for analyses.

We *diagnosed* LTLS based on the Cairo-Bishop definition of LTLS [4]. LTLS was considered present if levels of two or more serum values of the following were abnormal at presentation (as specified below) or if they changed by 25% within 3 days before until 7 days after cytotoxic therapy: SUA ≥ 8 mg/dL (476mol/L) or 25% increase from baseline; potassium (K) ≥ 6mg/dL (6.0 mmol/L) or 25% increase from baseline; phosphate (Phos) ≥ 4.5mg/dL (1.45 mmol/L) or 25% increase from baseline; albumin corrected calcium (Ca) ≤ 7mg/dL (1.75mmol/L) or 25% decrease from baseline, with two markers being abnormal within a 24-hour period. SUA was not included if urate oxidase was administered within previous 24 hours. Clinical TLS was defined as evidence of LTLS plus one or more of serum creatinine (SCreat > 1.5 x upper limits of normal), cardiac arrhythmia, seizure or sudden death. Acute kidney injury (AKI) was defined as an abrupt (within 48 hours) reduction in kidney function, defined as an absolute increase in serum creatinine ≥0.3 mg/dl (≥26.4 mmol/L), a percentage increase in SCreat ≥50% (1.5-fold from baseline in accordance with criteria established by the Acute Kidney Injury Network (AKIN) [5].

*Prediction* models of LTLS in AML utilized in the analyses included the Cairo (N.B., not synonymous with the Cairo-Bishop diagnostic criteria for LTLS referenced previously) [6], Surrey, West Sussex and Hampshire Cancer Network NHS [7] and Cancer and Leukemia Group B (CALGB) [8] and the proposed SUA criteria based on our previous findings in AKI [3]. The Cairo, NHS and the CALGB do not include SUA in their prediction models. The Cairo prediction model of LTLS in AML was developed by an international consensus expert panel and classifies patients according to WBC and LDH levels into low (WBC <25x10^9^/L and LDH <2x upper limits of normal), intermediate (WBC ≥25x10^9^/L and LDH ≥2x upper limits of normal) and high (WBC≥100x10^9^/L) risk. The NHS model classifies AML based only on WBC but with lower cutoff values: low, WBC <10x10^9^/L; intermediate, WBC 10–50x10^9^/L; and high risks, WBC ≥50x10^9^/L. It does not include LDH. CALGB classifies patients into favorable, intermediate and adverse groups based on remission outcomes for specific cytogenetic abnormalities and karyotype complexity. SUA criteria is based on our prior studies where SUA >5.5mg/dL was associated with a four-fold, SUA >6mg/dL with a six-fold, SUA >6.5mg/dL with an eight-fold SUA >7mg/dL with a forty-fold increased risk for AKI [3]. Accordingly, patients are classified as low (SUA <5.5mg/dL), intermediate (SUA >5.5mg/dL and <7mg/dL) and high (SUA ≥ 7mg/dL) risk for LTLS. SUA refers to baseline (pretreatment) values unless otherwise indicated. Waiver of consent approval for this retrospective analysis was obtained from the University of Florida Institutional Review Board. Only adult patients' data were included for analysis. Patient records/information was anonymized and de-identified prior to analysis.

### Statistical methods

Baseline patient characteristics are presented as mean ± SEM. Univariate logistic regression analysis was performed to determine the association of the known risk factors and incidence of LTLS, and also of pretreatment SUA and LTLS. The risk factors for LTLS included: age >60 years, primary vs. secondary AML, French-American-British (FAB) classification of subtypes, WBC, SCreat, markers on the leukemia cells, chromosome abnormalities, gene mutations, and prophylaxis and treatment protocols. These predictors were included in a multinomial logistic regression model to identify their independent effect on LTLS. We actively selected not to exclude any of the risk factors based on the known association with LTLS described previously and the risk of negative confounding. We also performed analysis that included only the variables that were significant in the unadjusted model. The strength of the association of each variable with LTLS was summarized by calculating an odds ratio (OR) and a corresponding 95% CI from the coefficients were estimated in the logistic regression models. Receiver-operating characteristic (ROC) curves were used to determine overall accuracy, as measured by area under the curve (AUC), to compare the ability of the different prediction models to predict LTLS. Statistical analyses were performed using SPSS, version 18.

## Results

### Baseline patient characteristics

Most patients were chronologically in their fifties and of Caucasian race with a preponderance of females **(Table 1)**. Serum uric acid at baseline was 5.1 mg/dL. Baseline SUA were measured at 2.8±0.1 days prior to initiation of induction therapy. SUA data were available in 57.3%, 31.6% and 21.3% of cases on days 1, 2 and 3 prior to initiation of treatment. SUA data were available in 89.1%, 90.7%, 95.6%, 94.5% and 93.9% of cases on days 0, 1, 2, 3 and 4 following initiation of treatment. Hypertension was present in a third of the patients. Most patients were diagnosed with primary (de novo) AML and received prophylactic therapy with the xanthine oxidase inhibitor allopurinol and bicarbonate containing intravenous hydration. Newly diagnosed patients (N = 139) were treated with an induction regimen consisting of cytarabine continuous infusion for 7 days plus either daunorubicin or idarubicin for 3 days. Patients with relapsed/refractory AML were treated with second line regimens, including CECA (cyclophosphamide, etoposide carboplatin and ARA-C); FLAG (fludarabine, cytarabine, G-CSF); FLANG (fludarabine, Ara-C, mitoxantrone); HiDAC (high-dose ARA-C); ATRA-IDA (Tretinoin plus idarubicin) and Mito-FLAG (mitoxantrone, fludarabine, cytarabine, G-CSF). Mean laboratory values, with the exception of LDH, were within normal limits. The most common FAB subtype was M0 **(Table 2).** CD34, linked to worse prognosis, was present in 54.1% of the patients. The cytogenetic risk profiles of the full cohort are shown in Table 2. The cytogenetic risk profiles of primary AML patients were similar: CALGB adverse, 27.9%; CALGB intermediate, 55.8%; and CALGB favorable, 16.3%. NPM1 (better prognosis) and FLT3 (poorer prognosis) gene mutations were identified only in a very small number of patients.

**Table 1:** Baseline patient characteristics.

Variables	Values
***Demographics***	N = 183
Age (years)	52.8±1.1
Caucasian race (%)	91.3
Female gender (%)	53
***Comorbidities***	
Hypertension (%)	34.4
Diabetes mellitus (%)	14.8
Coronary artery disease (%)	8.7
COPD (%)	3.3
***Medications***	
ACEI/ARB (%)	7.1
Diuretics (%)	20.8
***Tumor characteristics***	
Primary (de novo) AML (%)	76
Secondary AML (%)	24
***Treatment Regimen***	
Prophylaxis Allopurinol (%)	89.1
Urate oxidase (%)	8.2
Bicarbonate (%)	85.8
Induction (%) Full cohort	83.1
7+3 regimen	55.7
Other regimens	44.3
Re-induction (%)	16.9
***Laboratory***	
Serum uric acid (mg/dL)	5.1±0.2
Serum potassium (meq/L)	3.9±0.0
Serum phosphorus (mmol/L)	3.7±0.1
Serum calcium (mg/dL)	8.9±0.0
Serum creatinine (mg/dL)	0.9±0.0
Serum LDH (IU/L)	785.2±96.3
White blood count (x10^9^/L)	19.4±2.8
LTLS (%)	26.4
CLTLS (%)	5.4%

**Table 2:** AML subtypes, tumor markers and cytogenetic abnormalities.

Variables	N = 183
***FAB subtypes***	
M0 (%)	42.6
M1 (%)	6.6
M2 (%)	16.9
M3 (%)	15.3
M4 (%)	8.7
M5 (%)	8.7
M6 (%)	0
M7 (%)	1.1
***Tumor markers***	
CD34 (%)	54.1
*Cytogenetic abnormalities*	
CALGB adverse profile (%)	28.4
CALGB intermediate profile (%)	56.8
CALGB favorable profile (%)	14.8
***Gene mutations***	
NPM1 (%)	6
FLT3 (%)	6.6

### Incidences and risks of TLS

The incidence of LTLS in the full study cohort was 26.4% and that of clinical TLS (CTLS) was 5.4%. The incidence of CTLS was 7.1% when the more sensitive AKIN [5] definition was used instead of the criteria for AKI (>1.5x base SCreat) used in Cairo-Bishop definition of CTLS [4]. The incidence of LTLS and CTLS in patients with primary AML was 26.8% and 1.4%, respectively. The incidences of LTLS in the traditional high risk groups were as follows: age >60 years 28.8%, primary AML 26.8%, secondary AML 25%; WBC >100x10^9^/L 50%, LDH >2xULN 21.6%, CD34^+^ leukemias 16.3%, NPMI 27.2% and FLT3 gene mutation 33.3%. Significant effect of prophylactic sodium bicarbonate therapy on the incidence of LTLS could not be demonstrated (bicarbonate 24.8% vs. no bicarbonate 36%, p = 0.327). Analysis by prediction models yielded the following incidences of LTLS in each subgroups: Cairo, low-risk 31.4%, intermediate-risk 22.8% and high-risk 50%; NHS, low-risk 22.9%, intermediate-risk 26.2% and high-risk 32.6%; and CALGB, adverse 18.7%, intermediate 25.3% and favorable 44%. The SUA prediction model subgroups were associated with a 17.8% (low-risk), 21% (intermediate-risk) and 62.5% (high-risk) incidence of LTLS. Interestingly, we estimated that the incidence of LTLS would have been 17.4% if the definition of LTLS included only K, Phos and Ca but not SUA (LTLS_modified_).

The odds ratios (OR) were determined for the previously mentioned risk factors to predict LTLS. In the unadjusted model, CALGB favorable status, CD34 and pretreatment SUA (full cohort, low and high risk groups) were significant predictors of LTLS **(Table 3).** In the adjusted model, we actively selected not to exclude any of the risk factors based on the known association with LTLS described previously and the risk of negative confounding. In this model CD34 was the only significant predictor of LTLS (OR 0.3, CI_95%_ 0.1–0.6, p = 0.001). When we included only the variables that were significant in the unadjusted model, CALGB favorable status (OR 2.7, CI_95% 1.1_–6.5, p = 031), baseline SUA (OR 1.12, CI_95%_ 1.0–1.3, p = 0.048) and SUA high-risk group (OR 6.6, CI_95%_ 2.4–17.9, p<0.001) were significant predictors of LTLS.

**Table 3:** Univariate analysis of risk factors for AKI and LTLS.

Variables	AKI			LTLS		
	OR	CI_95%_	p-value	OR	CI_95%_	p-value
Age>60	4.23	1.6–11.1	0.003	1.21	0.6–2.4	0.577
***Tumor***						
Secondary AML, N = 44	1.69	0.6–4.4	0.294	0.91	0.4–1.9	0.812
***FAB subtypes***						
M0, N = 78	1.26	0.5–3.1	0.646	0.76	0.4–1.5	0.497
M1, N = 12	0.93	0.8–0.9	0.366	0.54	0.1–2.5	0.735
M2, N = 31	1.18	0.4–3.8	0.760	1.42	0.6–3.3	0.502
M3, N = 28	0.55	0.1–2.5	0.746	0.92	0.4–2.3	1.000
M4, N = 16	1.11	0.2–5.3	1.000	0.92	0.3–3.0	1.000
M5, N = 16	1.90	0.5–7.3	0.402	1.30	0.4–3.9	0.767
M6, N = 0						
M7, N = 2	0.99	0.9–1.0	1.000	1.04	0.9–1.1	0.068
***Pretreatment laboratory***						
WBC (full cohort), N = 183	1.00	_0.9–1.0_	0.497	1.00	_0.9–1.0_	0.390
WBC <10x10^9^/L, N = 95	1.27	0.9–1.7	0.082	0.94	0.7–1.2	0.603
WBC 10–50x10^9^/L, N = 43	1.02	0.9–1.1	0.627	0.98	0.9–1.0	0.477
WBC >50x10^9^/L, N = 15	0.99	0.9–1.0	0.825	1.00	0.9–1.0	0.449
WBC >100x10^9^/L, N = 6	0.98	0.9–1.0	0.617	0.99	0.9–1.0	0.943
SCreat, N = 183	3.67	1.5–9.0	0.005	1.42	0.7–2.8	0.313
SUA (full cohort), N = 183	1.22	1.1–1.4	0.003	1.12	1.0–1.2	0.042
SUA low risk, N = 113	0.52	0.2–1.2	0.162	0.33	0.2–0.6	<0.001
SUA intermediate risk, N = 38	0.88	0.2–3.4	0.856	1.22	0.5–3.1	0.663
SUA high risk, N = 32	3.54	1.3–9.4	0.012	7.26	3.2–16.6	<0.001
LDH, N = 145	1.00	0.9–1.0	0.468	1.00	1.0–1.0	0.930
LDH, 2xULN, N = 65	1.00	0.9–1.0	0.452	1.00	1.0–1.0	0.486
***Tumor markers***						
CD34, N = 99	0.75	0.3–1.8	0.528	0.32	0.1–0.6	<0.001
***Cytogenetics***						
CALGB (full cohort) = 169	0.81	0.4–1.7	0.593	1.83	1.1–3.2	0.031
CALGB adverse, N = 48	0.64	0.2–2.0	0.454	0.56	0.2–1.3	0.169
CALGB intermediate, N = 96	3.19	1.0–10.1	0.048	0.89	0.4–1.8	0.755
CALGB favorable, N = 25	0.00	0.0–0	0.998	2.62	1.1–6.3	0.032
***Gene mutations***						
NPM1, N = 33	0.42	0.1–2.5	0.347	1.00	0.1–5.1	1.000
FLT3, N = 35	3.06	0.3–29.7	0.336	0.87	0.2–3.4	0.322
***Prophylaxis***						
NaHCO3, N = 182	1.00	0.3–3.7	0.991	1.70	0.7–4.1	0.243
Allopurinol, N = 163	1.42	0.4–5.3	0.602	0.99	0.3–2.9	0.995
Urate oxidase, N = 15	0.31	0.1–1.1	0.066	1.50	0.4–5.5	0.561
***Treatment*** 7+3, N = 104	0.32	0.1–0.6	<0.001	1.10	0.6–2.1	0.777

Interestingly, we observed significant negative correlations between delta pre-post-treatment SUA and the incidence of LTLS (r = −0.32, p<0.001) and post-treatment peak K concentrations (r = −0.15, p = 0.042). To further investigate the effect of SUA on LTLS we subsequently restricted the analysis to patients with post-treatment increase in SUA (delta SUA) and used the definition of LTLS_modified_ discussed previously. Delta SUA was associated with a 1.4-fold increased risk for LTLS_modified_ (OR 1.43, CI95% 1.1–1.99, p = 0.032). In an adjusted model that included the previously referenced risk factors, SCreat (OR 3.1, CI_95%_ 1.3–7.2, p = 0.009) and high-risk SUA group were significant predictors of LTLS_modified._ (OR 2.8, CI_95%_ 1.1–7.1, p = 0.033).

### Incidence and risk of AKI

The incidence of AKI was 11.5% and 2.2% of the full cohort required dialysis therapy. Age >60 years was associated with the highest incidence of AKI (21.2%), followed by WBC >100x10^9^/L (16.6%), diagnosis of secondary AML (15.9%) and LDH 2x upper limits of normal (11.6%). Beneficial effects of prophylactic sodium bicarbonate therapy on the incidence of AKI could not be demonstrated (p = 1.000). We also determined the unadjusted ORs for AKI. Age >60 years, pretreatment SCreat, SUA (full cohort and high risk group) and 7+3 treatment regimen were significant predictors of AKI. In the adjusted model, age >60 years (OR 3.44, CI_95%_ 1.1 = 11.1, p = 0.040), pretreatment SCreat (OR 2.7, CI_95%_ 1.1–6.9, p = 0.035), CALGB intermediate status (OR 4.6, CI_95%_ 1.2–17.7, p = 0.024) and SUA (OR 1.3, CI_95%_ 1.1–1.5, p = 0.012) were significant predictors of AKI. We observed significant correlations between SUA and post-treatment peak SCreat in the high-risk groups of the prediction models investigated (Cairo, R^2^: 0.996 and CALGB, R^2^: 0.509). AKI was also associated with a four-fold (unadjusted OR 4.63, CI95% 1.8–11.8, p = 0.002; adjusted OR 3.97, CI_95%_ 1.5–10.5, p = 0.006) increased risk for LTLS adjusted for SUA.

### Comparison of combination pairs of SUA, K, Phos and Ca in the diagnosis of LTLS

**Fig 1:**
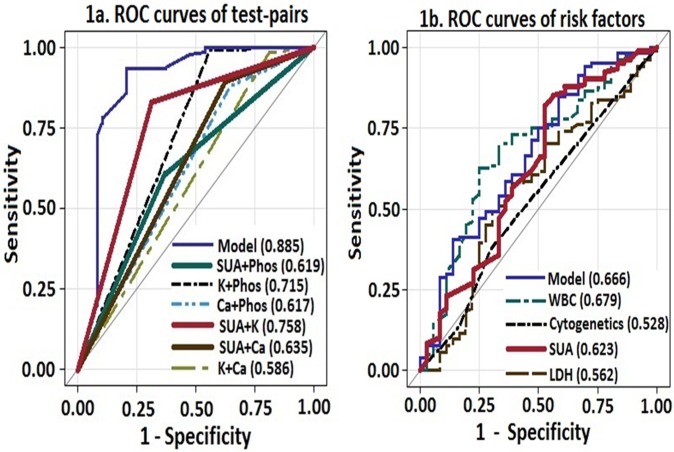
Comparison of ROC curves to predict LTLS: a) Test-pairs and b) Risk factors (cytogenetics, WBC, LDH and SUA).

The frequency of abnormal laboratory values were: SUA 27%, K 18%, Phos 47.5% and Ca 10.3%. The frequency of abnormal test-pairs for the diagnosis of LTLS was as follows: SUA-Phos 44.8%, SUA-K 30.6%, SUA-Ca 17.6%, K-Phos 12%, K-Ca 6% and Phos-Ca 18%. Since the Cairo-Bishop criteria for the diagnosis of LTLS is based on the change in levels of two or more serum values of SUA, K, Phos or Ca, we compared the diagnostic utility of the combination test-pairs of these variables using ROC curves **(Fig. 1A).** The combination pair of SUA-K had the best diagnostic performance, followed by K-Phos and SUA-Ca. The “model” curve displays the diagnostic performance when all the variables are included in the analysis.

### Comparison of prediction models of LTLS

**Fig 2:**
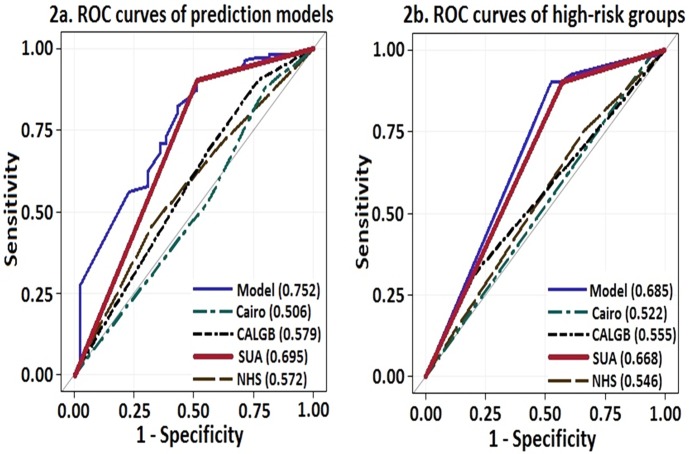
ROC curves of a) Prediction models (CALGB, Cairo, NHS and SUA) and b) High-risk groups of the prediction models.

We investigated the discriminatory ability of clinically important risk factors of LTLS by using AUC of the ROC curves **(Fig. 1B)**. The discriminatory ability of SUA was superior to LDH, cytogenetic profile, tumor markers and the combined model but not to WBC (AUC_WBC_ 0.679). However in comparisons between high-risk SUA and high-risk WBC, SUA had superior distinguishing capability (AUC_SUA_ 0.664 vs. AUC_WBC_ 0.520; p <0.001) to predict LTLS. We also compared the three prediction models described previously with SUA. We elected to include only the high-risk SUA group for comparison rather than the full SUA cohort due to the known properties of SUA to act both as an antioxidant and a pro-oxidant agent depending on its serum concentration, cellular location, and milieu. SUA demonstrated better performance than the prediction models (AUC_high-risk SUA_ 0.695, p<0.001; **Fig. 2A**). In direct comparison of high-risk groups of each prediction model, SUA again demonstrated superior performance than the prediction models (AUC _high-risk SUA_ 0.668, p = 0.001) in predicting LTLS, approaching that of the combined model (AUC 0.685, p<0.001; **Fig. 2B**).

### Length of stay and mortality

The mean LOS was 32.2±2.7 days (median 27 days). In patients with CD34+ or NPM1 or FLT3 gene mutations, the mean LOS was 33.8±3.6 days, 59.6±28.3 days and 60.0±25.7 days, respectively. The mean LOS according to the different prediction models were as follows: Cairo, low-risk 30.2±2.1 days, intermediate-risk 36.4±9.2 days and high-risk 41.5±8.5 days; NHS, low-risk 35.7±5.2 days, intermediate-risk 30.9±3.2 days and high-risk 33.4±6.8 days; CALGB, adverse 32.7±5.7 days, intermediate 32.9±4.7 days and favorable 33.9±3.6 days; and SUA, low-risk 36.5±4.2 days, intermediate-risk 25.8±2.2 days and high-risk 24.8±2.5 days. However, there were no significant correlations between LOS and the prediction models.

The overall all-cause mortality was 17.5% during the hospital stay. Patients who died belonged to the following groups per prediction model utilized: Cairo, low-risk 34.4%, intermediate-risk 28.1% and high-risk 3.1%; NHS, low-risk 34.4%, intermediate-risk 15.6% and high-risk 37.5%; CALGB, adverse 31.3%, intermediate 56.3% and favorable 3.1%; and SUA, low-risk 59.4%, intermediate-risk 28.1% and high-risk 12.5%. CD34+ and NPM1 and FLT3 gene mutations were present in 50%, 21.9% and 25% of the deceased, respectively. There were no significant correlations between mortality and the prediction models.

## Discussion

SUA is a catabolic product of nucleic acid, large amount of which is released into the circulation following spontaneous or treatment-induced lysis of tumor cells in AML. In this study we investigated the ability of pretreatment SUA to predict LTLS and directly compared its discrimination capacity to commonly utilized laboratory values, cytogenetic profiles, tumor markers and existing prediction models of LTLS in patients with AML. The major finding was that SUA had comparable predictive value as conventional prediction models and the combined model. SUA was associated with a significantly increased risk for LTLS and the effect size was six-fold higher in the high-risk group. Similar results have been reported wherein SUA >7.5mg/dL was associated with a 5.7-fold (CI_95%_ 2.6–12.7) increased risk for LTLS when compared to SUA<7.5mg/dL [9]. Our study differs from the previous study in that we utilized three distinct risk stratification levels, lower cut-off SUA values in each category and is based on previous experimental and clinical studies that demonstrated that SUA manifests pro-inflammatory properties at concentrations >5.5mg/dL [3,10–11]. Indeed, we demonstrated a progressively increased risk for LTLS and AKI respectively with higher SUA levels. In the direct comparison of risk factors of LTLS using ROC curves, SUA demonstrated superior performance to cytogenetic profile and LDH concentrations, but not to WBC. However, in high-risk groups, SUA was superior to WBC in predicting LTLS. The high-risk SUA group also demonstrated superior predictive performance than the Cairo, CALGB and NHS prediction models.

The CALGB favorable group was associated with a higher risk for LTLS compared to the intermediate and adverse groups, a reflection possibly of the greater chemo-responsiveness of the tumor cells. Although there were no significant correlations between LOS or mortality and prediction models, the observed trend towards decreased mortality in the CALGB favorable group is interesting since cytogenetics have been reported to be prognostic only in newly diagnosed AML patients. SUA was significantly correlated with post-treatment peak SCreat values in the high-risk groups of the prediction models. Similar relationships between SUA and SCreat have been demonstrated in cardiac surgery patients where crystal-dependent renal injury is not the predominant phenomenon [3,12]. These findings are clinically relevant considering the relationship between acute and chronic kidney diseases and the progression to end stage disease states [13].

Other important findings include the low incidence of CTLS and the relatively higher than expected incidence of LTLS than the previous report of 12% [9]. The development of CTLS has been reported to impact higher mortality rates from induction therapy in AML [9]. Another observation is the much lower than expected frequency of laboratory abnormalities compared to previous reports of 60–70% for SUA, K and Phos [9]. Although the diagnosis of LTLS is based on laboratory test-pair abnormalities, little data exist regarding their frequency. To that regard we provide data that SUA-containing test-pairs (SUA-K and SUA-Phos) were the most frequent laboratory abnormalities and also among the better performing diagnostic test-pairs (SUA-K, SUA-Ca, SUA-Phos).

Our observations also emphasize the emerging role of SUA in TLS via crystal-dependent and crystal-independent mechanisms [1–2,14]. Johnson et al have demonstrated in animal models that hyperuricemia in concentrations that do not result in crystal formation, causes renal vasoconstriction by activating the renin-angiotensin system, decreasing nitric oxide bioavailability, oxidant generation, upregulation of inflammatory mediators, proliferation of vascular muscle cells and inhibition of angiogemesis [15–17]. This resulted in a 40–60% decrease in renal blood flow and a 40–50% decrease in glomerular filtration rate (GFR) [10–11]. Lowering SUA with recombinant urate oxidase attenuated renal parenchymal damage [18]. Therefore, it is tempting to speculate that the observed negative correlations with changes in SUA and K concentrations and decrease in the incidence of LTLS were secondary to attenuated renal vasoconstriction and blunted decrease in renal blood flow and GFR from the lowering of SUA.

The strength of the current study is the direct comparison of laboratory values, cytogenetic profiles, tumor markers and prediction models of LTLS in a complex disease entity with relatively good sample size. Despite the inherent limitations of a retrospective analysis, the study provides ample evidence that SUA alone is relatively comparable and highly predictive for LTLS than the other prediction models. Each 1mg/dl increase in SUA was also associated with a 30% increased risk for AKI. AKI causes interruptions in therapy and substantially increases mortality in patients with cancer [19]. Therefore, it is critically important to accurately identify individuals who are at risk for LTLS and AKI. SUA alone is relatively comparable and highly predictive for LTLS than other expensive models that includes tumor markers and cytogenetic profiles.
